# Cholangiocarcinoma of the Common Bile Duct Presenting as Empyema of the Gallbladder: A Rare Case Report

**DOI:** 10.7759/cureus.59865

**Published:** 2024-05-08

**Authors:** Sanjay M Khaladkar, Neeha A Jhala, Aryaman N Dhande, Prajakta P KirdatPatil, Suhas M

**Affiliations:** 1 Radiodiagnosis, Dr. D. Y. Patil Medical College, Hospital & Research Centre, Dr. D. Y. Patil Vidyapeeth, Pune, IND

**Keywords:** mrcp, common bile duct, cholangiocarcinoma, gallbladder, empyema

## Abstract

Cholangiocarcinoma of the common bile duct (CBD) presenting as empyema of the gallbladder is a rare entity that poses a risk of septicemia, septic shock, peritonitis, and abscess formation. This case report describes an elderly female presenting with pain in the right hypochondrium, a positive Murphy’s sign, and a history of regurgitation and constipation. It highlights the value of imaging in the early diagnosis of this rare presentation of underlying malignancy. The most common cause of empyema of the gallbladder is acute cholecystitis, followed by gallbladder neck obstruction by a calculus. This report discusses the important role of imaging supported by clinical history, examination, laboratory tests, and histopathological findings to diagnose this rare presentation of empyema of the gallbladder as a complication of underlying cholangiocarcinoma. Additionally, it briefly discusses the change in the management line for cholangiocarcinoma patients with complications such as gallbladder perforation and septicemia. The study concludes that the possibility of underlying bile duct malignancy cannot be overlooked in patients with similar symptoms, particularly among the elderly.

## Introduction

Empyema of the gallbladder occurs when pus or purulent material fills the distended gallbladder lumen. This condition often arises as a complication of acute cholecystitis and obstruction of the gallbladder neck by a calculus. It typically manifests as pain and tenderness in the right hypochondrium, accompanied by a positive Murphy's sign observed in the right subcostal area and a clinical history of fever [1]. These symptoms are further supported by an elevated total leukocyte count in the laboratory report.

Cholangiocarcinoma refers to malignant epithelial tumors originating from the biliary tree, excluding the gallbladder or the ampulla of Vater. It typically manifests in individuals in their late sixties and ranks as the third most common primary hepatobiliary malignancy following hepatocellular carcinoma (HCC) and gallbladder cancer [2]. In the Western world, its annual incidence ranges from 0.35 to 2.00 per 100,000 individuals. Over the past three decades, this incidence has steadily increased to 0.1-0.6 per 100,000 in countries such as the United States and the United Kingdom. However, certain regions like Japan and northern India exhibit a notable clustering of cholangiocarcinoma cases [3].

While the disease often presents late, common signs and symptoms include pain in the right hypochondrium, fatigue, weight loss, and symptoms of obstructive jaundice such as yellowish skin discoloration, pruritus, and clay-colored stools.

Among the three types of cholangiocarcinoma, the lesion arising from the cystic duct and proximal common bile duct (CBD) (perihilar cholangiocarcinoma) can sometimes obstruct bile drainage through the cystic duct. This obstruction results in stasis of bile with superimposed infection caused by *Escherichia coli*, *Streptococcus faecalis*, *Klebsiella*, and anaerobes like *Bacteroides* and *Clostridia*, leading to empyema of the gallbladder [1]. This case underscores the importance of a comprehensive understanding of the unique clinical presentation of cholangiocarcinoma. While it rarely manifests as empyema of the gallbladder, such presentations can potentially lead to severe complications, including gallbladder perforation and septicemia. Early diagnosis, particularly in elderly patients, is crucial for improving patient prognosis. 

As not many cases of cholangiocarcinoma of the proximal CBD presenting as empyema of the gallbladder have been reported, this case highlights the importance of considering malignancy as a potential differential diagnosis in elderly patients presenting with symptoms of empyema of the gallbladder.

## Case presentation

A 67-year-old female presented at a hospital in Central India with pain in the right hypochondrium, fever, a history of constipation, regurgitation, and decreased appetite over the past three months. On clinical examination, a palpable lump was detected in the right hypochondrium, along with a positive Murphy’s sign in the subcostal region. The patient denied experiencing yellowish discoloration of the skin, pruritus, or passage of clay-colored stools. Additionally, there was no reported history of other medication intake or comorbidities.

Given the clinical presentation, the patient was advised to undergo radiological and pathological assessments. Acute cholecystitis was suspected based on the presented symptoms.

**Figure 1:**
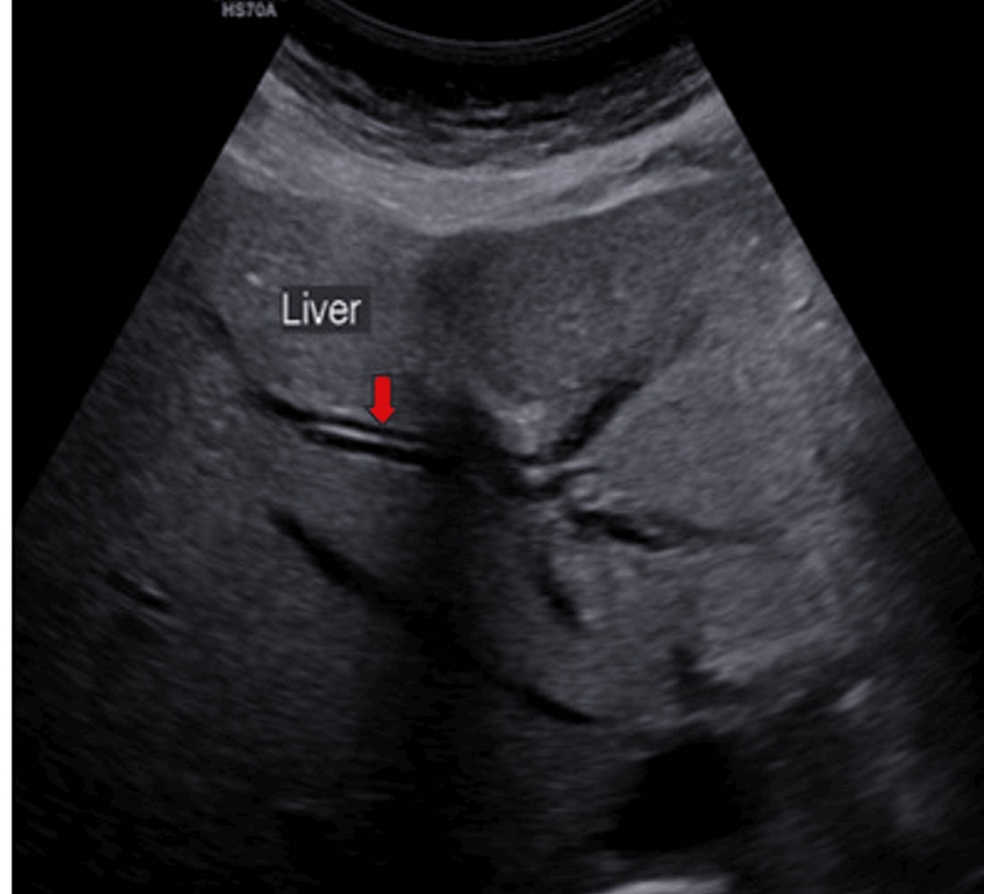
Ultrasonography image of the liver showing IHBR dilatation (red arrow). IHBR: Intrahepatic biliary radicle

**Figure 2:**
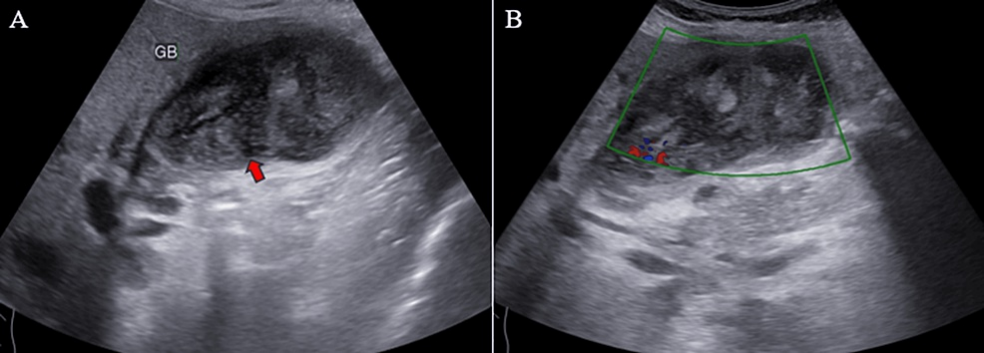
Ultrasonography images demonstrate heterogeneously hypoechoic content (red arrow) in the gallbladder (A) and absent vascularity within the content on color Doppler (B). The peripheral vascularity observed is artifactual. GB: Gallbladder

**Figure 3:**
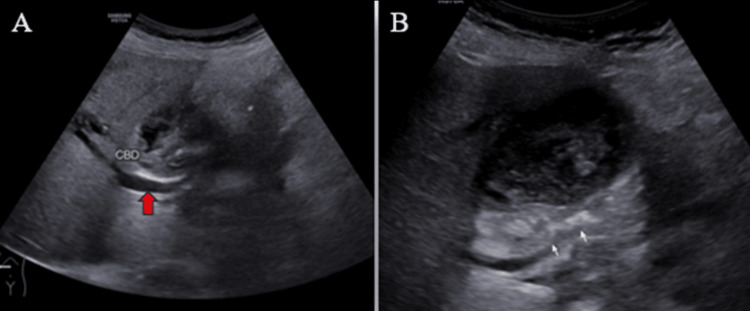
Ultrasonography images show the dilated proximal CBD (red arrow) (A) and a heterogeneously hypoechoic lesion (white arrow) in the mid-CBD, accompanied by narrowing of this part of the CBD (B). CBD: Common bile duct

**Figure 4:**
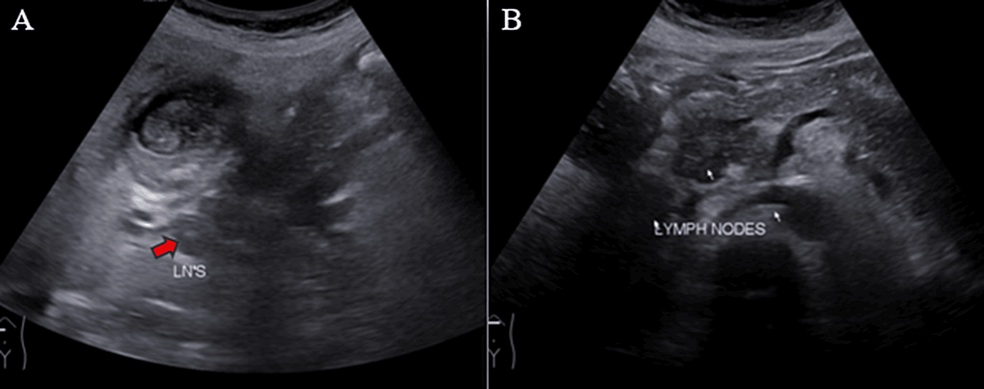
Ultrasonography images reveal enlarged necrotic lymph nodes in the periportal region (red arrows) (A) and retroperitoneal region (white arrows) (B). LN'S: Lymph nodes

Ultrasound (US) examination revealed bilobar intrahepatic biliary radicle (IHBR) dilatation (Figure 1), and the gallbladder appeared overdistended with large, heterogeneously hypoechoic content devoid of vascularity on color Doppler (Figure 2). The gallbladder wall appeared thickened, measuringapproximately 4 mm. Furthermore, the proximal CBD appeared mildly dilated, measuring 8 mm, with a heterogeneously hypoechoic lesion within the midportion causing its narrowing (Figure 3). Multiple subcentimetric to enlarged necrotic lymph nodes were also observed in the porta-hepatis, periportal, and retroperitoneal regions (Figure 4).

These findings were suggestive of an infective etiology, likely indicative of acute cholecystitis with thick purulent content in the gallbladder and a mass lesion in the mid-portion of the CBD.

**Figure 5:**
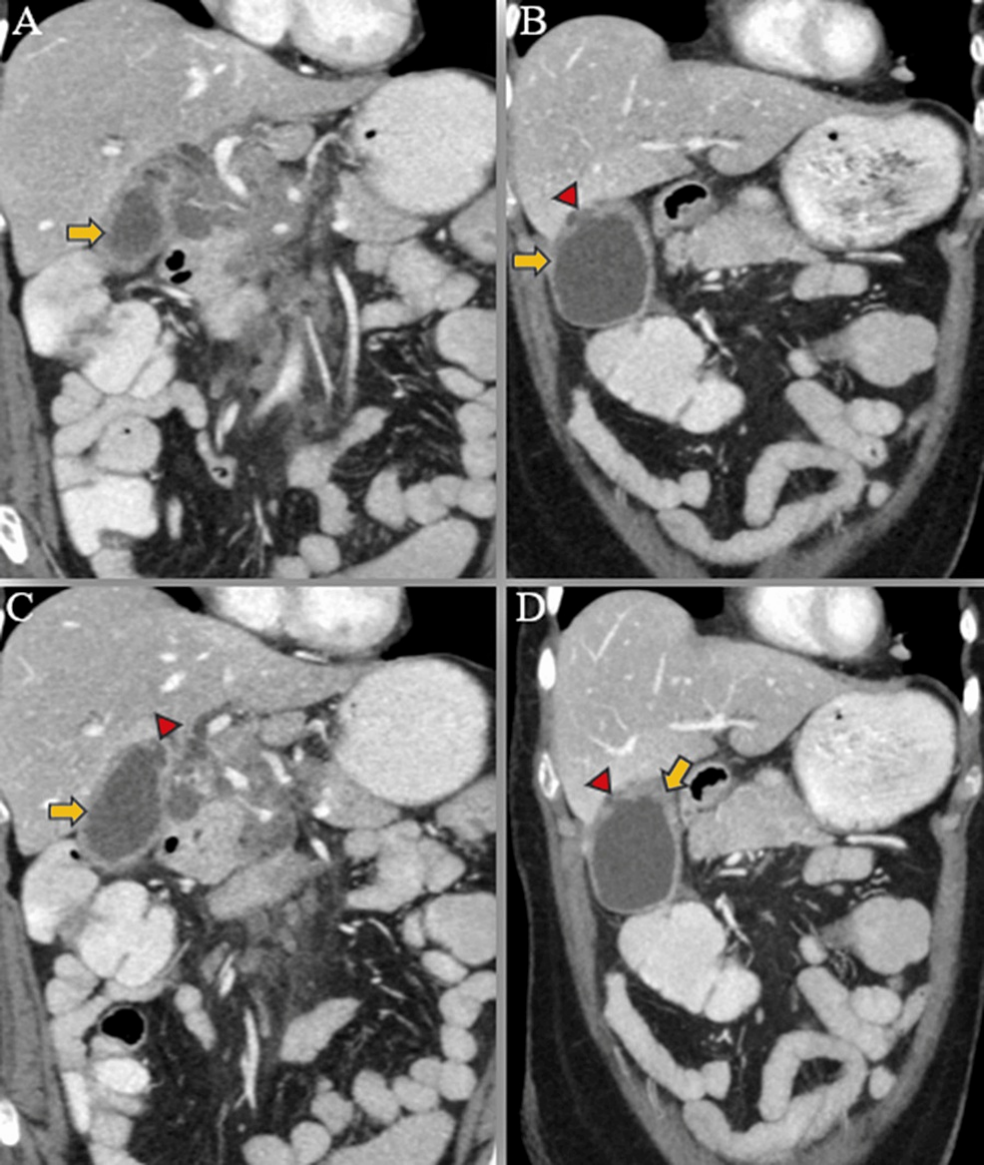
Post-contrast coronal reformatted CT images show a thickened wall of the gallbladder (yellow arrows) exhibiting post-contrast enhancement (A-D), with a defect (red arrowheads) observed in the wall of the body of the gallbladder, accompanied by a hypodense pericholecystic fluid collection (B-D).

**Figure 6:**
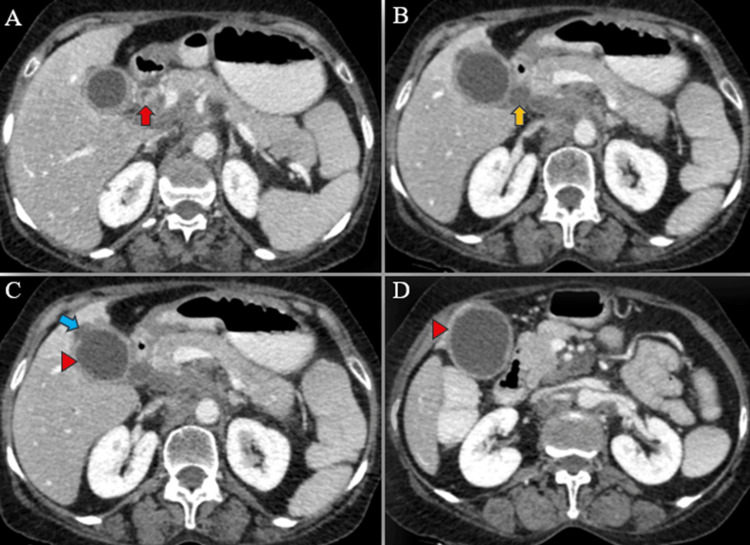
Post-contrast axial CT images reveal circumferential thickening of the mid-CBD (red arrow) (A) along with dilation of the proximal CBD (yellow arrow) (B). Additionally, a defect in the gallbladder body region (blue arrow) (C) is accompanied by a surrounding hypodense pericholecystic collection and thickened gallbladder wall (red arrowheads) (C-D). CT: Computed tomography; CBD: common bile duct

A triphasic computed tomography (CT) scan of the abdomen and pelvis revealed mild IHBR dilatation in both the right and left hepatic lobes. The gallbladder was overdistended, with mild wall thickening (4 mm), exhibiting post-contrast enhancement. Additionally, a small 5 mm defect was identified in the body region, accompanied by a well-defined hypodense fluid density lesion measuring approximately 13 x 6 mm adjacent to it. No radiodense calculus was detected. The proximal CBD showed dilation (8 mm) with mild eccentric wall thickening in the mid-CBD (maximum thickness 5 mm), showing post-contrast enhancement. The remainder of the CBD appeared normal in caliber and wall thickness (Figures 5-6).

**Figure 7:**
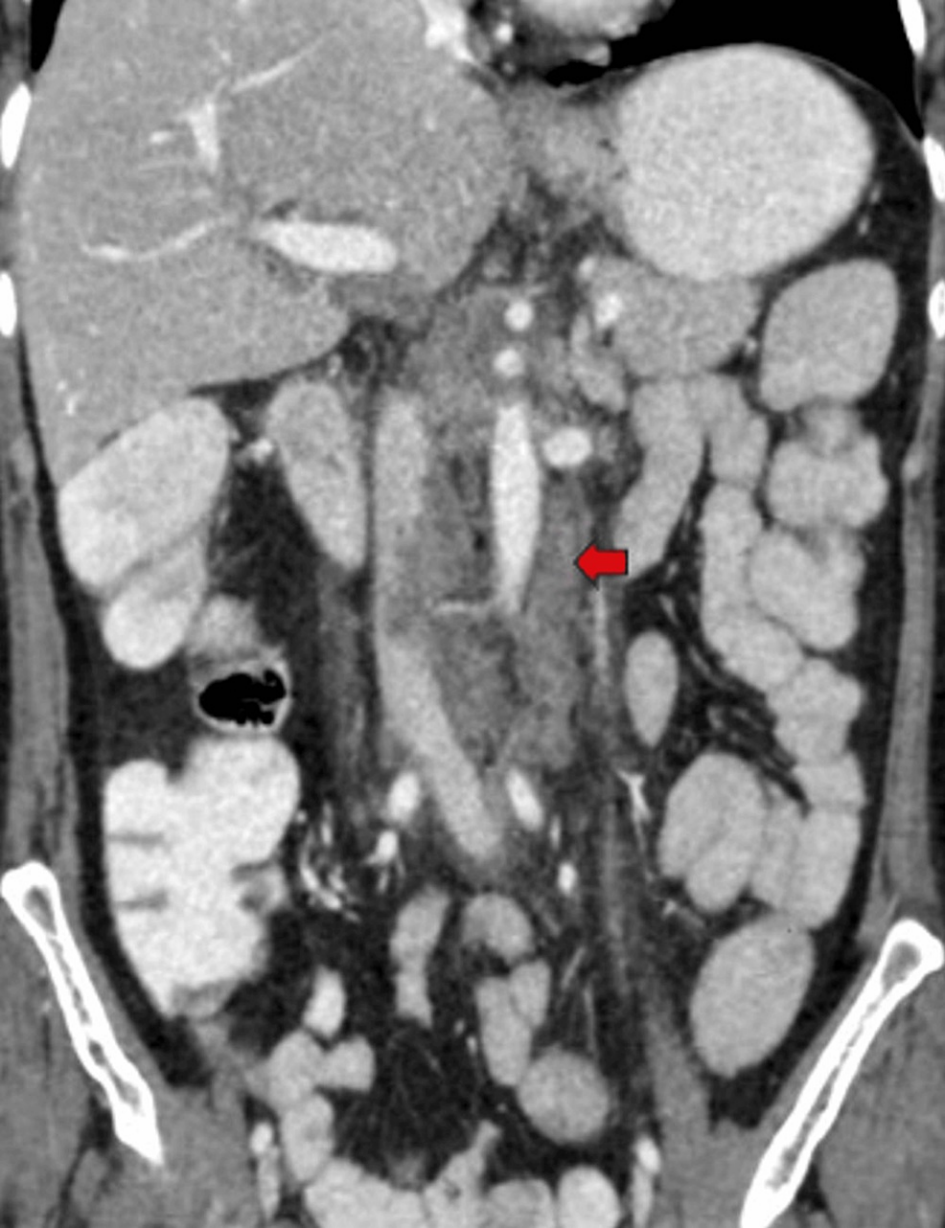
Post-contrast coronal reformatted CT image show enlarged and conglomerated lymph nodes seen in the para-aortic (red arrow) and aorto-caval regions with central hypodense nonenhancing necrotic areas. CT: Computed tomography

Furthermore, multiple lymph nodes of variable sizes, demonstrating mild heterogeneous post-contrast enhancement, were noted at the porta hepatis, peripancreatic region adjacent to the pancreatic head, celiac region, aorto-caval region, pre-aortic, and left para-aortic region, with the largest measuring approximately 18 x 35 mm (anteroposterior x transverse) at the porta hepatis (Figure 7). Both adrenal glands appeared normal, and there was no evidence of ascites. The rest of the abdominal organs exhibited normal appearances.

**Figure 8:**
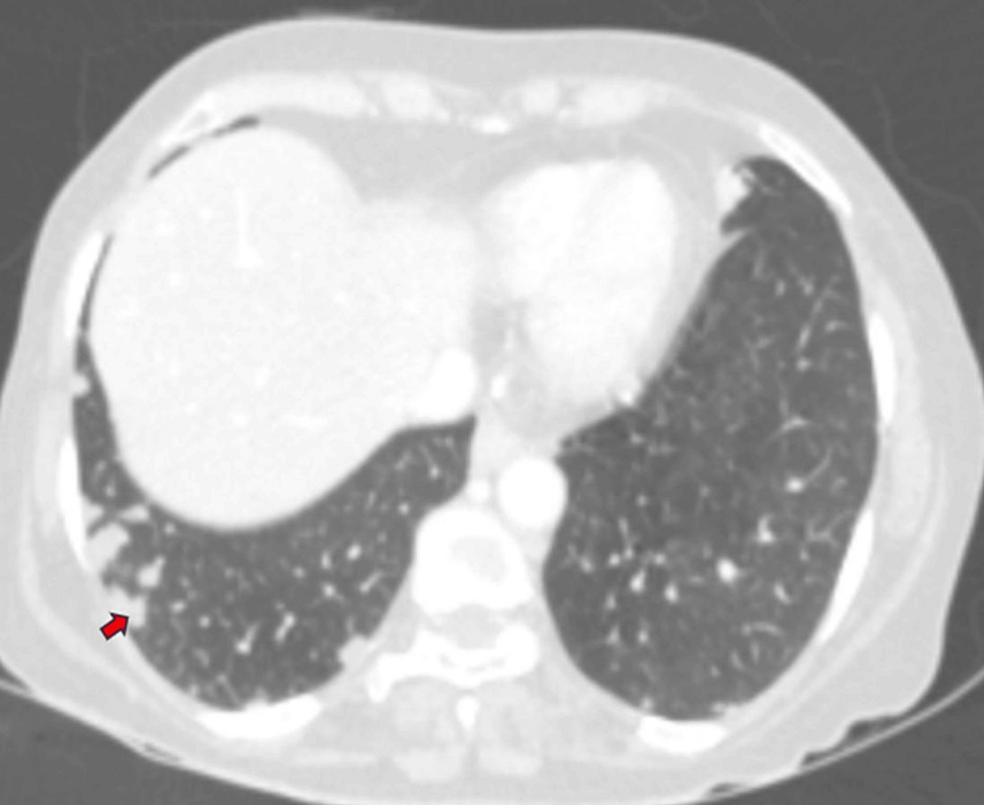
CT axial lung window image of the base of thorax showing multiple well-defined subpleural pulmonary nodules (red arrow). CT: Computed tomography

Additionally, multiple well-defined soft tissue density nodular lesions, ranging in size from 2 to 10 mm, were observed in both lower lobe basal segments, predominantly peripheral and subpleural in location, with mild heterogeneous post-contrast enhancement (Figure 8). Visualized bones appeared normal, without evidence of osteolytic or osteoblastic metastases.

Overall, these findings suggested a neoplastic mass originating from the mid-CBD, leading to an overdistended gallbladder, cholecystitis, and contained perforation in the body region, resulting in mild proximal obstructive biliary dilatation. Additionally, metastatic lymph nodes were identified at the porta hepatis and retroperitoneum, along with multiple lung metastases.

**Figure 9:**
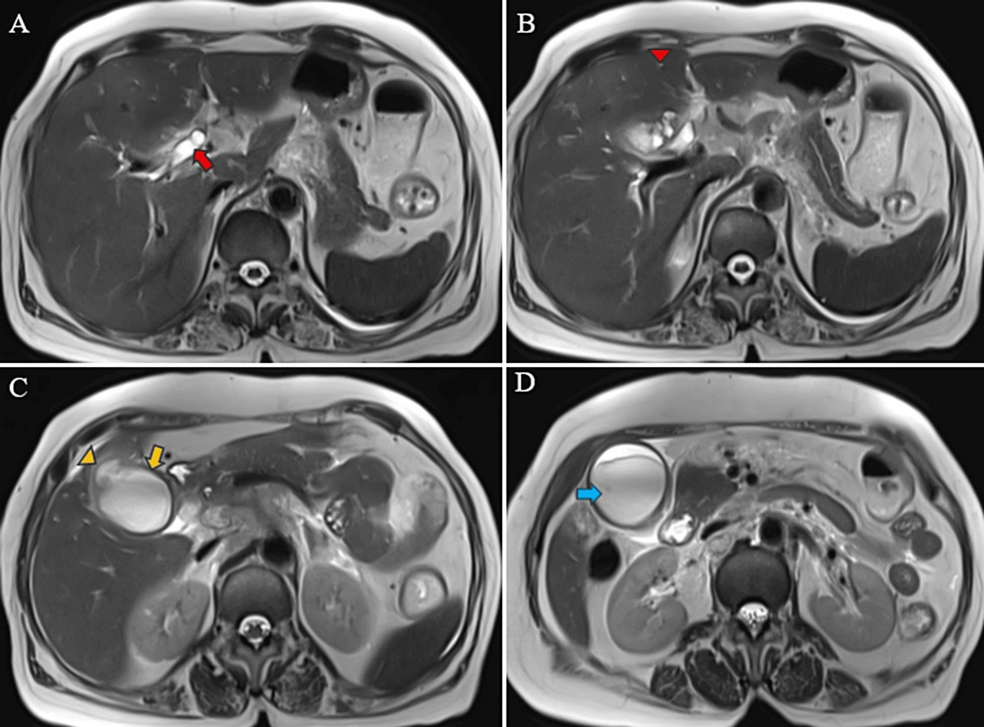
MR abdomen T2WI axial images reveal dilated right and left hepatic ducts (red arrow) with prominent intrahepatic bile radicles (IHBR) (red arrowhead) (A, B). A thickened gallbladder wall (yellow arrow), a defect in the gallbladder body region (yellow arrowhead), and a pericholecystic collection (C) are observed. Additionally, a distended gallbladder with a collection within it is seen (blue arrow) (D). MR: Magnetic resonance; T2WI: T2-weighted imaging

**Figure 10:**
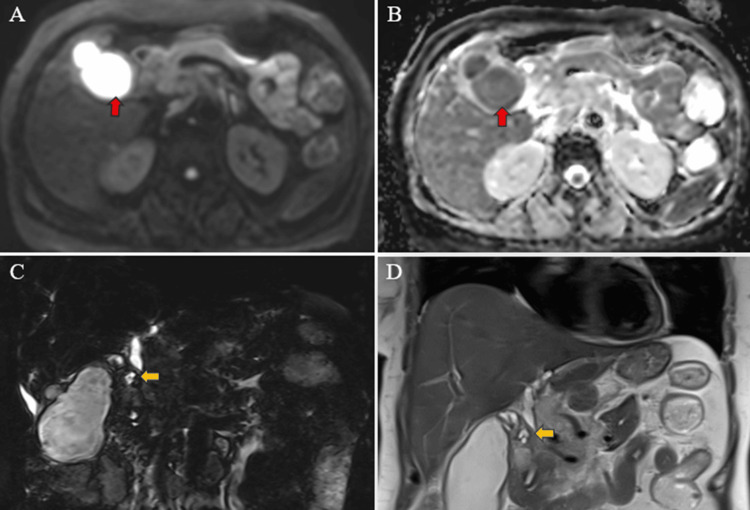
MR abdomen and MRCP images depict the gallbladder collection and pericholecystic collection (red arrow) showing restricted diffusion on DWI (A) and corresponding low ADC values observed in the same gallbladder and pericholecystic collection (red arrow) (B). Additionally, narrowing in the mid-CBD (yellow arrow) with dilated proximal CBD is evident on T2WI SPACE (C) and T2WI HASTE (D) images. MR: Magnetic resonance; MRCP: MR cholangiopancreatography; T2WI: T2-weighted imaging; DWI: diffusion-weighted imaging; ADC: apparent diffusion coefficient; SPACE: sampling perfection with application optimized contrast using different flip angle evolution; HASTE: half-Fourier single-shot turbo spin-echo

Magnetic resonance (MR) abdomen imaging with MR cholangiopancreatography (MRCP) revealed an overdistended gallbladder with circumferential wall thickening measuring 4 mm. Bile appeared hypointense on T2-weighted imaging (T2WI), indicative of sludge or thick material, with diffusion restriction on diffusion-weighted imaging (DWI) and low apparent diffusion coefficient (ADC) values. A 7 mm defect was noted in the body region, with T2 hyperintense pericholecystic fluid collection demonstrating diffusion restriction adjacent to the body. Mild dilatation of the IHBR was observed in both the right and left lobes. The CBD measured 8 mm in diameter, with narrowing of the mid-CBD due to circumferential wall thickening measuring 4 mm. The remainder of the CBD appeared normal in caliber (Figures 9-10).

**Figure 11:**
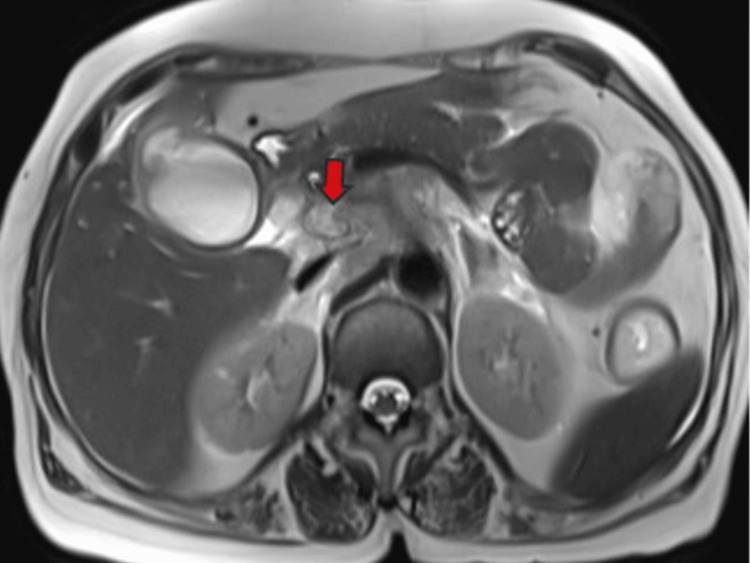
MR abdomen T2WI axial image showing a heterogeneous appearing enlarged necrotic pre-caval lymph node (red arrow). MR: Magnetic resonance; T2WI: T2-weighted imaging

Multiple enlarged lymph nodes were noted at various locations, including the porta hepatis, peripancreatic region adjacent to the pancreatic head, celiac region, aorto-caval region, pre-aortic, left para-aortic region, and retrocaval region (Figure 11). These lymph nodes exhibited T1WI hypointensity and T2WI heterogeneous hyperintensity, with central necrotic areas of variable sizes. Some lymph nodes demonstrated diffusion restriction on DWI and appeared coalescent. The pancreas appeared normal. Visualized lower lobes of both lungs exhibited well-defined T2WI hyperintense lesions ranging in size from 2 to 15 mm, with peripheral and subpleural distribution.

These findings were suggestive of a stricture in the mid-CBD, likely neoplastic (cholangiocarcinoma), resulting in proximal biliary obstructive dilatation. Additionally, the gallbladder exhibited changes consistent with empyema and contained perforation. Multiple metastatic lymph nodes were observed in the retroperitoneum and porta hepatis, along with multiple lung metastases.

Endoscopic retrograde cholangiopancreatography (ERCP) revealed marked narrowing in the mid-18 mm of the CBD. Cholangiogram demonstrated dilation of the CBD (8 mm) proximal to the CBD stricture in the mid-CBD. Biliary brush cytology was obtained from the stricture site. A biliary sphincterotomy was performed, and a guidewire was successfully passed into the right system. The stricture was dilated using 9 Fr and 12 Fr Soehendra biliary dilation catheters. Subsequently, a 10 Fr × 7 cm double-pigtail CBD stent was placed, resulting in the free flow of bile. The diagnosis of cholangiocarcinoma causing obstructive biliary dilatation was confirmed.

The histopathological report of endoscopic ultrasound (EUS)-guided biopsy from the hilar lymph node and the stricture in the mid-CBD revealed a tumor composed of small nests and glands lined by low cuboidal to columnar epithelium with hyperchromatic pleomorphic nuclei, scanty eosinophilic cytoplasm, and prominent nucleoli. The stroma exhibited desmoplastic changes with chronic inflammatory infiltrate. These histological findings indicate adenocarcinoma with metastases.

Laboratory investigations revealed abnormalities including a raised total leucocyte count (TLC), elevated liver enzymes, increased levels of C-reactive protein, elevated erythrocyte sedimentation rate, and elevated carcinoembryonic antigen (CEA) levels .

**Table 1:** Laboratory investigations LFT: Liver function test; ALT: alanine transaminase; AST: aspartate transaminase; ALP: alkaline phosphatase; HBsAg: Hepatitis B surface antigen; RFT: renal function test; BUN: blood urea nitrogen; CBC: complete blood count; WBC: white blood cells; ESR: erythrocyte sedimentation rate; CRP: C-reactive protein; CA 19.9: carbohydrate antigen 19.9; CEA: carcinoembryonic antigen; PT: prothrombin time; INR: international normalized ratio

Test	Observed value	Reference range
LFT		
Total bilirubin	0.8 mg/dL	0.3-1.0 mg/dL
Direct bilirubin	0.1 mg/dL	0.0-0.3 mg/dL
Indirect bilirubin	0.2 mg/dL	0.1-0.7 mg/dL
ALT	171 U/L	7-56 U/L
AST	132 U/L	5-40 U/L
ALP	227 U/L	35-104U/L
Total proteins	5.8 g/dL	6.0-8.3 g/dL
HBsAg	Non-Reactive	-
Hepatitis C virus antibodies	Non-Reactive	-
RFT		
Serum creatinine	0.8 mg/dL	0.6-1.3 mg/dL
Blood urea nitrogen	11.8 mg/dL	7-20 mg/dL
CBC		
WBC	15,500/μL	4000-10,000/μL
Absolute neutrophil count	13,640/μL	2000-7000/μL
ESR	85 mm per Hour	<10 mm per hour
CRP	21 mg/L	<10 mg/L
Serum Amylase	60 U/L	40-140 U/L
Serum Lipase	45 U/L	25 – 150 U/L
CA 19.9	16.09U/mL	0-37U/mL
CEA	70.19 ng/ml	0-5 ng/ml
Coagulation profile		
PT	11 seconds	10.0-14.0 seconds
INR	1.1	0.8-1.2

Based on the findings described above, the patient was diagnosed with cholangiocarcinoma presenting as empyema of the gallbladder with contained gallbladder perforation and distant lymph nodal and pulmonary metastases. The patient was advised further management; however, she refused any intervention and surgical treatment. Consequently, she was treated conservatively with antibiotics and antacids.

## Discussion

Cholangiocarcinoma encompasses three primary types classified by the tumor, node, metastasis (TNM) staging, each dependent on its location within the biliary system: intrahepatic, hilar (or perihilar), and distal cholangiocarcinoma. The intrahepatic variant primarily involves the intrahepatic biliary radicles. In contrast, the hilar/perihilar variant extends to the cystic duct insertion, affecting the secondary biliary radicles. The distal variant is characterized by the involvement of the duct distal to the cystic duct. Hilar/perihilar cholangiocarcinoma, also known as Klatskin tumor, stands as the most common subtype within this classification scheme.

Macroscopically and radiologically, cholangiocarcinoma can be classified according to the Liver Cancer Study Group of Japan [4]. The classification includes three main types: mass-forming, periductal infiltrating, and intraductal. Mass-forming cholangiocarcinomas are characterized by a defined mass within the liver parenchyma. Periductal infiltrating cholangiocarcinomas extend longitudinally along the bile duct, often resulting in peripheral bile duct dilation. Intraductal cholangiocarcinomas proliferate within the lumen of the bile duct. This classification system provides valuable insights into the distinct features of different types of cholangiocarcinomas [4].

In the Western world, primary sclerosing cholangitis stands as the predominant cause of cholangiocarcinoma, whereas parasitic liver diseases have shown a notable association with the condition in the Eastern world [5]. With an insidious onset and often manifesting late in the disease course, patients diagnosed with early-stage extrahepatic cholangiocarcinoma face a daunting five-year survival rate of merely 18%. Even if the cancer has disseminated to regional lymph nodes, the survival rate remains at 18%. However, in cases where distant spread is evident, the survival rate drastically plummets to a mere 2% [6].

Patients with cholangiocarcinoma typically present with pain in the right hypochondrium, fatigue, weight loss, and symptoms of obstructive jaundice such as yellowish discoloration of the skin, pruritus, and clay-colored stools. Laboratory investigations often reveal raised liver enzymes and elevated tumor marker levels of carbohydrate antigen 19-9 (CA 19.9) and CEA [7]. The treatment and prognosis of cholangiocarcinoma depend on the resectability of the tumor and its staging. The only potential cure is through surgical resection if the tumor is resectable and has negative tissue margins. However, the majority of cases present as unresectable disease due to distant spread. In such cases, the gemcitabine/cisplatin combination remains a standard treatment according to the guidelines from the National Comprehensive Cancer Network (NCCN) [8].

Empyema of the gallbladder is a common complication of acute cholecystitis and obstruction of the gallbladder neck by a calculus. Rarely, in cases of hilar or distal cholangiocarcinoma, drainage of pus through the cystic duct can become obstructed, leading to empyema. Stasis of bile, combined with infections caused by bacteria such as *Escherichia coli*, *Streptococcus faecalis*, *Klebsiella*, and anaerobes such as *Bacteroides* and *Clostridia*, further contributes to the development of empyema [1]. If not promptly drained or removed, the gallbladder wall can become edematous, tense, and necrotic, eventually perforating. In rare instances, fistulous communication may occur, potentially leading to the involvement of the overlying anterior abdominal wall and subsequent septicemia

In our patient, cholangiocarcinoma in the proximal CBD obstructed bile flow, leading to empyema of the gallbladder, which further complicated into a contained gallbladder perforation. This acute onset of symptoms may result in a misdiagnosis of acute empyematous cholecystitis, thereby masking the underlying malignancy.

Treatment typically involves open or laparoscopic cholecystectomy or decompression of the distended gallbladder, either laparoscopic or under radiological guidance. Antibiotic therapy is administered until fever and septicemia subside. Cholangitis occurs in approximately 30.6% of cases in patients with biliary tract cancer [9].

In patients with comorbid conditions and unresectable tumors, such as likely cholangiocarcinoma, empyema can be treated by percutaneous transhepatic cholecystoduodenal stents [10]. Endoscopically placed stents can also be inserted via the transpapillary route [11]. They can also be placed across pre-existing cholecystoduodenal fistulas [12].

## Conclusions

Cholangiocarcinoma often presents insidiously, with symptoms such as pain in the right hypochondrium, weight loss, and signs of obstructive jaundice. However, in rare instances, it may manifest as empyema of the gallbladder, leading to complications such as perforation, septicemia, and peritonitis. In elderly females presenting with symptoms of toxemia and biliary tract obstruction, the possibility of cholangiocarcinoma should be considered. Cases have been reported where cholangiocarcinoma was incidentally found during laparotomy for acute cholecystitis or cholelithiasis. Imaging plays a crucial role in facilitating early diagnosis of cholangiocarcinoma, enabling prompt initiation of treatment and improving the patient's five-year survival rate. With our thorough understanding of cholangiocarcinoma and empyema of the gallbladder separately, this case highlights the relationship between the two and underscores the importance of recognizing empyema as a potential presenting feature of underlying malignancy. Such recognition can significantly improve the prognosis of the patient.
